# Predictors of Preschool Neurodevelopmental Outcomes and Trajectories in a South African Birth Cohort

**DOI:** 10.21203/rs.3.rs-10971095/v1

**Published:** 2026-09-18

**Authors:** Kirsten A Donald, Marilyn T. Lake, Simone R Williams, Catherine J Wedderburn, Susan Malcom-Smith, Andrea M Rehman, Nadia Hoffman, Tiffany Burd, Heather J Zar, Dan J Stein

**Affiliations:** 1 Department of Paediatrics and Child Health, Red Cross War Memorial Children’s Hospital, University of Cape Town, Cape Town, South Africa; 2 Neuroscience Institute, University of Cape Town, Cape Town, South Africa; 3 Department of Psychiatry and Mental Health, University of Cape Town, South Africa; 4 ASCENT lab, Department of Psychology, University of Cape Town, Cape Town, South Africa; 5 MRC Statistics & Epidemiology group, London School of Hygiene & Tropical Medicine, London, United Kingdom; 6 SAMRC Unit on Risk and Resilience in Mental Disorders, University of Cape Town, Cape Town, South Africa; 7 SAMRC Unit on Child & Adolescent Health, University of Cape Town, Cape Town, South Africa

**Keywords:** brain development, cognition, functional neurodevelopmental outcomes, pre-school

## Abstract

**Background:**

Early childhood is a critical period for brain development, yet many children in low- and middle-income countries face adversities that put them at risk of not reaching their developmental potential. To address gaps in understanding factors which predict developmental outcomes at 5 years in a South African birth cohort, this research assessed which early-life factors shaped developmental outcomes at 5 years and trajectories between 2–5 years.

**Methods:**

728 mother–child dyads from the Drakenstein Child Health Study, were followed from the antenatal period through 5 years. Neurodevelopmental outcomes (cognition, receptive and expressive language, fine motor skills) were measured between 2–5 years using standardized instruments. Random forest models identified key predictors of each domain. Longitudinal trajectories from 2–5 years were identified using latent class growth analysis; and random forest modelling examined predictors of class membership.

**Results:**

Fifty theoretically motivated predictors of neurodevelopment accounted for over 90% of variance across neurodevelopmental domains at 5 years, in this South African birth cohort. Cross-validated models retained 9 for neurocognition, 15 key predictors for receptive language, 3 for expressive language and 1 for fine motor skills. The greatest variance explained was observed for neurocognition (23%), with modest variance explained for other domains. Critical predictors of neurocognition in this cohort included socioeconomic status indicators, maternal gravida and childhood trauma, sex, early anthropometry, and parenting practices. Predictors that demonstrated overlap between neurocognition and receptive language domains were socioeconomic status indicators, maternal gravida and parenting practices. Receptive language also included domain-specific predictors including maternal mental health, intimate partner violence, social support and childbirth anthropometry. Expressive language was best explained by 3 predictors, including in-utero HIV exposure, maternal smoking and gravida, whilst fine motor skills were best predicted exclusively by child sex. Trajectory analysis revealed 8.8–16% of children demonstrated developmental catch-up between 2–5 years. Across both univariate and multivariate classification models, 8 top-ranked predictors discriminated recovery-class membership; where child anthropometry, parenting practices and household crowding were shared predictors across models.

**Conclusion:**

Alongside factors predicting neurodevelopment variance at age 5-years, trajectory analysis identified developmental catch-up among a group of at-risk children, with maternal and environmental factors predicting this recovery trajectory.

## 2. Introduction

To date, epidemiological data suggest that approximately 250 million (43%) children from low- and middle- income countries (LMICs), under the age of 5 years are at risk of adverse neurodevelopmental outcomes (1). During early development, the brain is especially susceptible to adverse exposures that can have lasting downstream consequences on functional neurodevelopment and subsequent brain function (2). Conversely, protective factor encounters during early childhood development can promote resilience in children and can positively impact neurodevelopment, (3), leading to favourable long-term neurological outcomes (4) and in some instances reduce susceptibility to neuropsychiatric disorders that may arise during adulthood (5, 6).

A growing body of literature supports the notion that exposure to health, environmental and psychosocial risk factors during early development can adversely affect functional neurodevelopmental outcomes of motor, cognition, and language. Research shows that impaired neurocognitive outcomes in children are associated with early exposure to a variety of risk factors including prenatal alcohol (7, 8), polydrug exposure (9), household and community violence (10, 11), maternal anaemia (3), maternal depression during pregnancy (12) and perinatal exposure to infectious diseases including HIV (13, 14) or cytomegalovirus (15). Similar risk factors have also been implicated as risk factors for adverse language (16, 17) or motor (18, 19) developmental outcomes in children. Furthermore, research from participants enrolled in the Drakenstein Child Health Study (DCHS), a South African birth cohort study, have demonstrated that exposure to the risk factors of antenatal maternal posttraumatic stress disorder (20) or HIV exposure (13, 14) can contribute to poor child neurodevelopmental outcomes in the early years.

Existing literature similarly demonstrates how neurodevelopmental outcomes in children may be beneficially impacted by protective factors, including good nutrition and breastfeeding, clean and safe homes, nurturing environments, and parents with good physical and mental health. Children who were breastfed and performed better across the full range of developmental domains, had more active maternal bonding and a lower risk of internalising behavioural impairment at 6 years of age when compared to their non-breastfed counterparts (21, 22). In Korea, another birth-cohort study demonstrated that breast feeding for more than 3 months was associated with higher child neurodevelopmental scores in communication and problem-solving domains at 2 years, expressive language at 3 years and vocabulary and language inference at 8 years, when compared to children who had breastfed for three months or less (23). Furthermore, at 2 years of age in the DCHS birth cohort we reported better child developmental outcomes across domains of cognition, receptive and expressive language, and motor development associated with higher maternal education and socioeconomic status (3). We also found higher birth weight to be significantly associated with greater scores and lower odds of delay for all neurodevelopmental domains^3^.

Collectively these studies highlight associations between both risk and protective factors, and functional neurodevelopmental outcomes in children. However, much of the existing published work reports outcomes at discrete timepoints and fewer incorporate models that include Antenatal and postnatal exposures (including post-infancy nutrition, health and stimulation) may impact children during the pivotal preschool period (24). Some research has explored longitudinal neurodevelopmental trajectories in high income settings and particular for clinical risk groups such as children born prematurely or with very low birth weight. A birth cohort study in Taiwan identified three cognitive and motor trajectories (normal, deteriorating and persistently delayed) in the cohort of infants between 6 to 36 months of age (25). Key findings included low birthweight and low parental socio-economic status as factors associated with the deteriorating trajectories, while prolonged hospitalisation and major brain insults were associated with more stable, but persistently delayed trajectories. A further study from the same region described three similar motor trajectories in a cohort of preterm infants all with VLBW (26). A Japanese population-based birth-cohort study, identified five classes of neurodevelopmental trajectories (high normal, normal, low normal, delay and markedly delayed) in infants between 1 and 24 months of age, where male biological sex, younger gestational age, lower birthweight, and lower maternal education were found to predict odds of membership in the markedly delayed class (27). Another cohort study in Brazil found increases in cognitive and motor scores in infants, assessed three times over four months, to be associated with protective factors such as higher socioeconomic status, higher parental education as well as parenting practices (28).

To our knowledge, functional neurodevelopmental outcomes related to risk and protective factors have mainly been studied in infants, while little is known about outcomes in older pre-school children and the which antenatal and early childhood risk and protective factors may predict outcomes at these later ages or neurodevelopmental trajectories in LMIC settings.

There has been a growing interest in applying machine learning to neurodevelopmental research in early childhood, to identify children at risk of not reaching their optimal developmental potential (29,30,31). Machine learning offers an opportunity to identify risk and protective patterns that may be clinically relevant amongst complex and large datasets (32, 33, 34). Despite its promise, the use of machine learning in approaches in this field remains limited in LMICs, where risks to early child neurodevelopment are amongst the highest (29,35,36).

The present study aimed to identify the top-ranked predictors of cognitive, receptive and expressive language, and fine motor skills from birth through 5-years - using a supervised machine learning technique, random forest modelling. This combined approach leverages use of predictive models; with random forest modelling used to identify and rank the most important prenatal and postnatal predictors. Together, this approach aids in reduction of complex datasets with large numbers of candidate factors to identify most relevant predictors. Using univariate and multivariate latent class growth modelling, the study furthermore set out to identify classes of trajectory performance across domains between 2–5 years of age and to explore factors that may predict performance trajectories.

## 3. Methods

### 3.1 Study design and setting

This study was performed as part of in the Drakenstein child health study (DCHS). The DCHS is a multi-dimensional longitudinal, observational birth cohort study in the peri urban Drakenstein district in Paarl, Western Cape, South Africa. Communities within the Drakenstein municipality, particularly those served by the Mbekweni or TC Newman clinics are characterised by a high prevalence of socioeconomic risk factors, including low levels of employment and educational attainment (37); psychosocial risk factors, including high rates of maternal depression, drug and alcohol abuse and intimate partner and community violence; and infectious disease risk factors, including high rates of HIV and TB. Given that high risk factor prevalence within the Drakenstein district is representative of many other communities within South Africa and other LMICs, the DCHS aimed at investigating child health and developmental outcomes in the context of environmental, infectious, nutritional, genetic, psychosocial, maternal, and immunological factors, especially prevalent and relevant in the context of South Africa and other similar (LMICs).

### 3.2 Participants

Pregnant women were recruited from two primary health care clinics, Mbekweni (serving a predominantly black African community) and TC Newman (serving a mixed ancestry community). Mothers were enrolled in the DCHS at 20 to 28 weeks’ gestation while attending routine antenatal care and were prospectively followed. Women were eligible for the study if they were 18 years or older, between 20–28 weeks gestation, planned attendance at one of the two recruitment clinics and intended to remain in the area. Maternal and Child initial sample size: Between 5 March 2012 and 31 March 2015, 1225 pregnant women were enrolled into the DCHS; 88 (7.2%) mothers were lost to follow up antenatally, had a miscarriage or a stillbirth. In total, 1137 women gave birth to 1143 live infants (4 sets of twins and 1 triplets) from 29 May 2012 to 3 September 2015. Enrolment criteria were broad to ensure that the cohort would be representative of the general population. Ethical approval was obtained from the Faculty of Health Science, Human Research Ethics Committee at the University of Cape Town (HREC 401/2009; 525/2012) and by the Western Cape Provincial Health Research Committee (REF:2011RP45). Mothers provided informed consent at enrolment and were re-consented annually. Consent was done in mother’s preferred language: English, Afrikaans or isiXhosa.

### 3.3 Data Collection Procedures

#### 3.3.1 Sociodemographic and maternal characteristics

**Figure 1 |:**
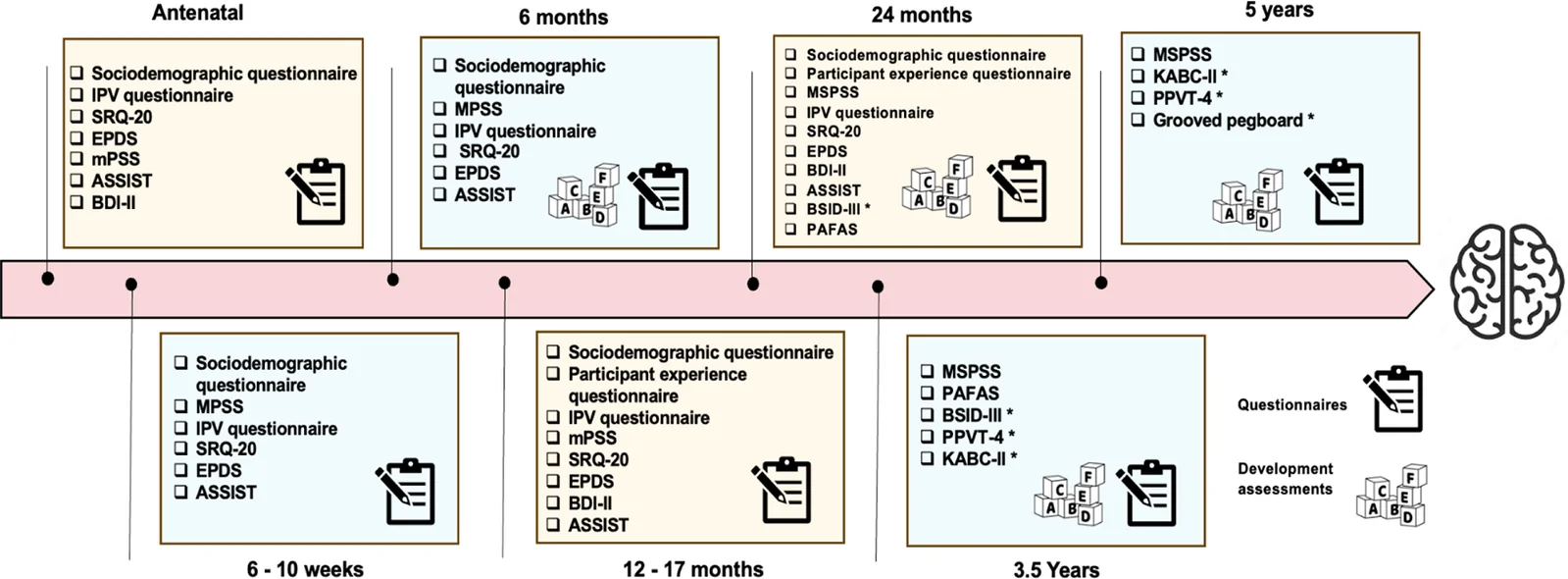
Timeline of sociodemographic characteristics and functional neurodevelopmental outcomes acquired from antenatal to 5 years. *Neurodevelopmental assessments. mPSS, Multidimensional Scale of Perceived Social Support; PAFAS, Parenting and Family Adjustment Scale; ASSIST, Alcohol, Smoking and Substance Involvement Screening Test questionnaire; EPDS, Edinburgh Postnatal Depression Scale; BDI-II, The Beck Depression Inventory; mPSS, modified PTSD symptom depression scale, Clinical Administered PTSD Scale and SRQ-20, the Self Reporting Questionnaire; BSID-III, Bayley Scales of Infant and Toddler Development, third edition.

Biomedical, environmental, psychosocial, demographic, physical data were collected at various time points ranging from 6–10 weeks to 5.5 years (Figure 1). Information on infant birth and feeding methods were collected antenatally, 6–14 weeks, 6 months and 9 months timepoints (37). Visits included a clinical examination, interviews on self-report questionnaires and assessment measures. As previously reported (37,38), asessments included: the sociodemographic questionnaire, to establish socio-economic status based on employment status, educational level and household resources and amenities; the Multidimensional Scale of Perceived Social Support (MSPSS), a self-report questionnaire on perceptions of social/capital support from family, friends and/or partners; The Parenting and Family Adjustment Scale (PAFAS), a parent-report measure assessing parenting practices, parent-child relationship quality, parental emotional adjustment and teamwork, and family relationship; the Alcohol, Smoking and Substance Involvement Screening Test (ASSIST) questionnaire, self-report questionnaire on maternal alcohol, smoking and substance use; the modified PTSD symptom depression scale (mPSS), an interviewer administered diagnostic instrument to measure PTSD; the Self Reporting Questionnaire (SRQ-20), a self-report questionnaire assessing psychological distress, including symptoms of depressive and anxiety disorders; The Beck Depression Inventory (BDI-II), self-report measure of depressive symptoms; Edinburgh Postnatal Depression Scale (EPDS), questionnaire on maternal depression, which includes 5 separable latent classes derived from longitudinal trajectories between pregnancy through to 18 months postpartum using group-based trajectory modelling as previously described (39); the Intimate Partner Violence (IPV) Questionnaire, self-report adapted for the DCHS to assess lifetime and recent exposure to emotional, physical and sexual IPV. Also included were 5 IPV combination latent classes (across emotional, physical and sexual IPV domains) derived from latent class growth modelling from pregnancy through to 24 months postpartum as described by Barnett and colleagues (2018) (40).

#### 3.3.2 Functional neurodevelopmental outcomes

Neurodevelopmental outcomes were assessed at different timepoints by trained research assistants and assisted by translation where needed (Figure 1), these methods have been described in detail elsewhere (41). At each timepoint, all children who were active in the DCHS and living in area were invited for the assessment visit. Children were all accompanied by a parent/guardian. The Bayley’s Scales of Infant and Toddler Development, third edition (BSID-III) was used to assess functional neurodevelopmental outcomes at 24 months of age, and for fine motor performance at 3.5 years. The BSID-III objectively measured outcomes in domains of cognition, receptive and expressive language and fine and gross motor development. Raw BSID-III scores were calculated as the sum of individual items passed by the child in each domain and were converted to an age-adjusted standardised Z-scores using normative data from a reference population (42).

At 3.5 and 5 years of age, the Kaufman Assessment Battery for Children, second edition (KABC-II) was used to assess neurodevelopmental outcomes including general cognitive function, measured by the Nonverbal Index (NVI), which was derived from subtests that do not rely on language, allowing for an assessment of general cognition independent of language abilities. The NVI consists of the KABC-II Face recognition task (working memory), KABC-II Conceptual thinking (Problem solving), KABC-II Hand movements (working memory and motor), and KABC-II Triangles (visual-spatial memory/processing) subtests. In addition, expressive language was assessed using the KABC-II Expressive vocabulary subtest. (43). All neurodevelopmental scores on BSID-II and KABC-II are represented as age-adjusted scaled scores according to a norm-referenced population.

The Peabody Picture Vocabulary test, version 4 (PPVT-4) was used to assess receptive language at 3.5 and 5 years. The PPVT-4 measures receptive vocabulary and is designed for use in a wide age range (ages 2.5 to 90 years). The examiner reads out a word, and then the child responds by pointing to the picture they think corresponds to the word the examiner has given. The test is untimed but takes approximately 10 – 15 minutes. The PPVT-4 has excellent test-retest reliability and internal consistency (44). The PPVT-4 has been translated into both Afrikaans and isiXhosa (45).

The Grooved Pegboard (Lafayette Instrument Company, Inc.) was used at 5 years to assess motor control. The assessment tests manipulative dexterity in the dominant and non-dominant hands. The total completion time for each trial is recorded. The Grooved Pegboard has excellent test-retest reliability for both hands and relatively strong concurrent validity in a 3–9-year-old sample (46). The Grooved Pegboard has been used successfully in South African samples (47). Raw scores were used for PPVT-4 and Grooved pegboard. For longitudinal analyses, scores from different measures of the same neurodevelopmental domain were standardised into z-scores to allow for comparability over time

### 3.4 Statistical analysis

**Figure 2 |:**
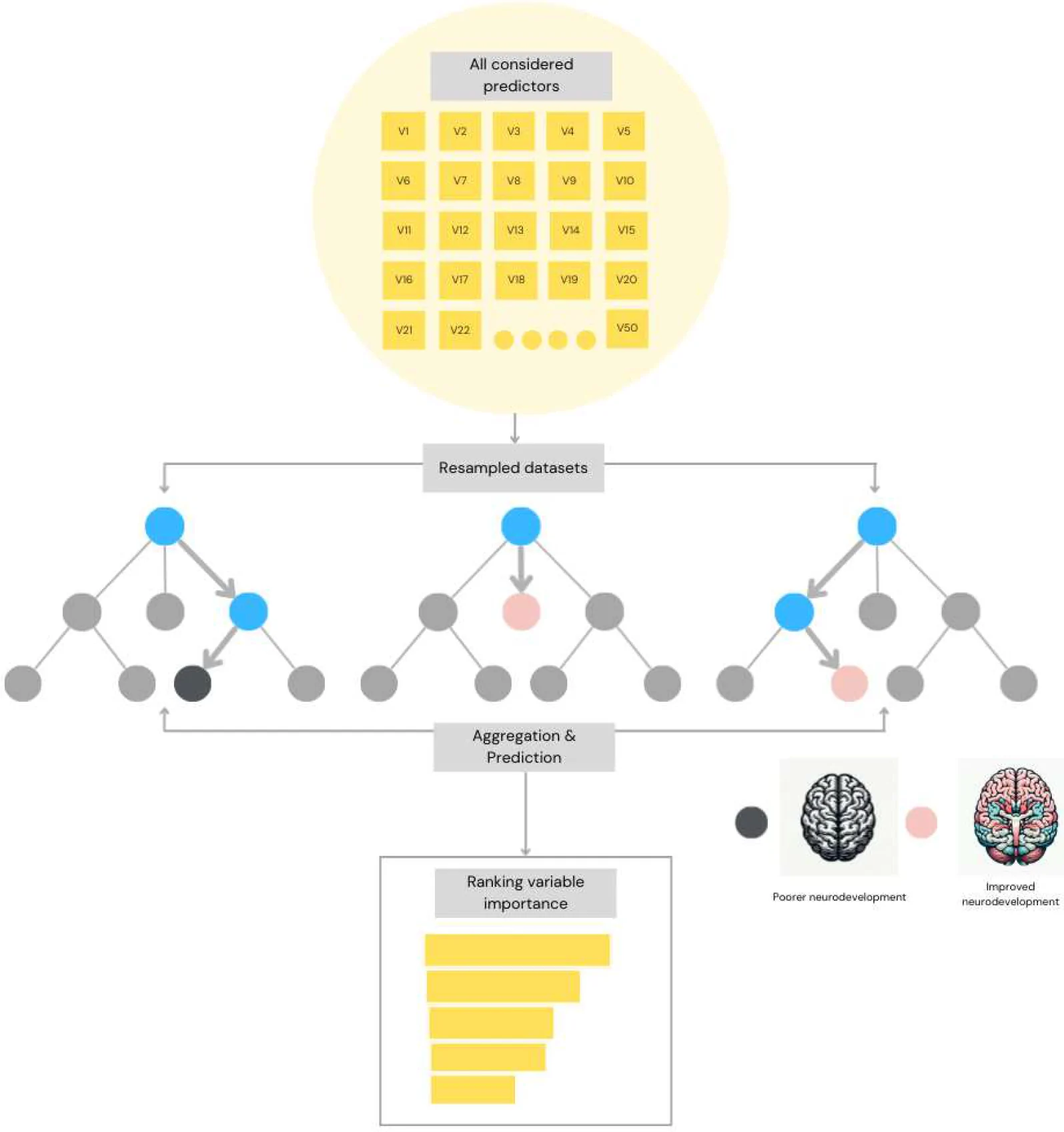
Conceptual framework of Random Forest Modelling. A total of 50 predictor variables were first identified based on their theoretical relevance to neurodevelopment. Random forest modelling was then applied to training data. For each tree, participants were randomly sampled with replacement (using bootstrapping). Within each tree (three decision trees shown for illustrative purposes), a random subset of predictors was selected at each node, ensuring that each tree was trained on different data. The random forest (illustrated by multiple decision trees) then combined/aggregated predictions from individual decision trees. In addition to prediction, random forest modelling estimated variable importance or contribution of each predictor to model accuracy. The major strength in random forest modelling lies in its ability to highlight the most relevant risk and protective factors within a large set of considered predictors.

Maternal and child sociodemographic characteristics were presented as mean (sd) for continuous variables while categorical data were expressed as absolute frequencies (%). In the cross-sectional outcome analyses of cognitive performance at 5 years, random forest regression modelling was utilised to identify the most important predictors from the antenatal period to postnatal age two years, selected from a pool of 50 candidate predictors (Table 1) for (1) cognition, (2) receptive language, (3) expressive language and (4) fine motor skills at 5 years. Random forest (RF) modelling is a popular supervised machine learning algorithm that aggregates results across multiple decision trees and can be used for both classification and regression problems (Figure 2). Given the large number of possible risk and protective factors that may predict developmental outcomes, RF modelling was utilised to highlight the most important risk and protective predictors for cognitive and developmental outcomes (1) given its capacity to handle high dimensional data (3) and it’s utility in ranking of the most important predictors, offering more flexibility than traditional regression models.

For the analyses of predictors of cognition and development at 5 years, using the *caret* r package (48), data was first divided into 80% training and 20% testing data sets, ensuring similar distributions of neurodevelopmental outcomes across both. 5-fold cross-validation in combination with grid search was applied to random forest regression models to tune parameters that optimized prediction performance where targeted parameters included the number of predictors included at each split (*mtry*), number of trees (*ntrees*) and minimum sample size at final nodes (*nodesize*). Model predictive performance was evaluated according to Root mean squared error (RMSE) and R-squared predicted estimates using test data. Missing predictor data was imputed using the random forest imputation algorithm in *missForest* (49) r package. All 50 candidate predictors were included in the random forest models, where predictors importance was ranked according to mean decrease accuracy. Post-hoc 5-fold cross-validation was conducted to select the optimal number of predictors to retain, where model comparisons were based on test data predictive performance metrics including RMSE and R-squared.

For the longitudinal outcome analyses, univariate latent class growth modelling (LCGM) was conducted to identify subgroups with distinct trajectories in cognition, receptive language, expressive language, and fine motor domains across 2–5 years. The optimal number of latent classes was selected based on the Bayesian Information criterion, entropy, and minimal class size of 5%. In addition, a multivariate LCGM was also conducted to examine whether shared latent class trajectories emerged across the 4 neurodevelopmental domains. A similar random forest modelling process to that described for cross-sectional outcome analyses was applied to the longitudinal analyses. In this case, random forest classification models were used to predict latent class membership, with the outcome variable represented by latent growth class assignment derived from univariate and multivariate LCGMs. Parameter tuning was consistent with that described under regression, but classification performance was evaluated using Receiver Operating Characteristic Area Under the Curve (ROC-AUC). In both cross-sectional and longitudinal outcome analyses, final models were selected based on a combination of predictive accuracy and parsimony.

A sensitivity analysis was conducted to test the plausibility of the Missing-at-Random (MAR) assumption for imputed data. Random Forest Modelling was utilised to predict missingness of each variable and model performance was evaluated using the ROC-AUC. In the absence of formal thresholds for discriminating Missing-At-Complete-Random (MCAR) from MAR using ROC-AUC values, estimates were interpreted against conventional discrimination thresholds proposed by Hosmer and Lemeshow (2000). ROC-AUC estimates <0.60 were used to indicate low predictability of missingness from observed data, more consistent with MCAR, while estimates between ≥0.60 and <0.70 were used to indicate moderate predictability, and estimates ≥0.70 to indicate stronger predictability of missingness, more consistent with MAR assumption.

## 4. Results

### 4.1 Participant sociodemographic characteristics

**Figure 3 |:**
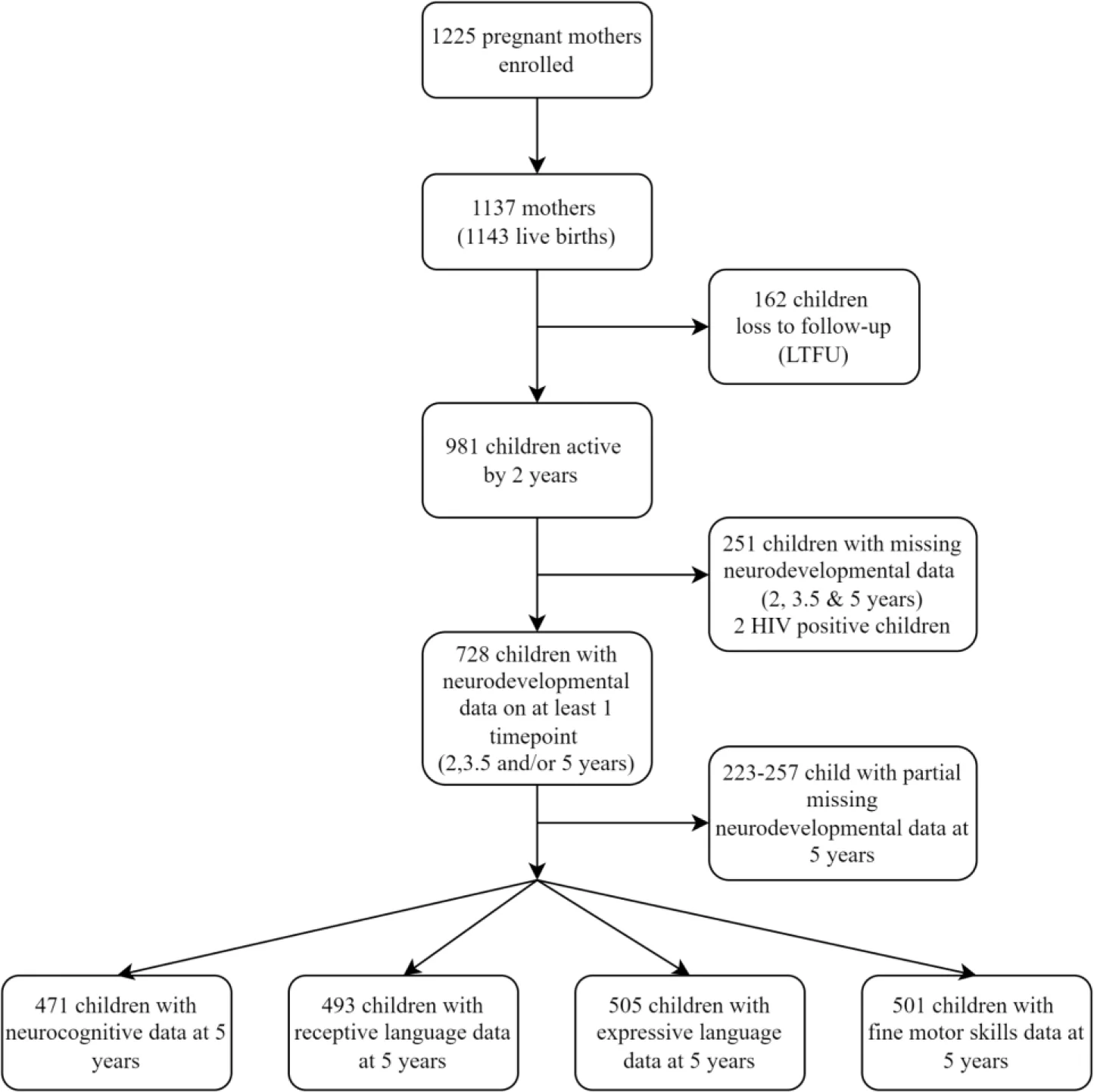
Flow diagram of DCHS participants at timepoints from enrolment to 5 years of age

The analysis included 728 mother-child dyads with available neurocognitive data from the DCHS. Figure 3 depicts the study flow from enrolment of mothers in pregnancy through to children with developmental assessments at 5 years of age. Within this group, 49% of the children were female, 14% were born preterm (less than 37 weeks), 177 (24%) experienced hospitalization in the first two years, 23% were born to mothers living with HIV, but were uninfected themselves, and 17% of mothers had documented antenatal anaemia (Table 1). In terms of nutritional and growth factors, 17% of children were exclusively breastfed for 6 months, children started solid food at an average age of 4 months, and height-for-age and weight-for-height z-scores were −1.06 (sd: 1.18) and 0.32 (sd: 1.25), respectively at 24 months.

Of the mothers included in the sample, 40% were married or cohabiting with a partner; 37% completed secondary schooling and above; and 182 (25%) were employed antenatally (Table 1).

Almost all households (99%) had a postnatal household monthly income of greater than R1000 on at least one visit over the postpartum period. Most households (90% and above) had access to electricity both antenatally and postnatally, while approximately 70% had access to piped water. In this sample, 61% of mothers reported above threshold childhood trauma exposure, 47% had experienced some postnatal intimate partner violence and approximately 20% reported having above threshold psychological distress both antenatally and postnatally (Table 1). The prevalence of antenatal and postnatal maternal depression was 24% and 27% respectively, based on the Edinburgh Postnatal Depression Rating Scale. Of the mothers included, 100 (14%) reported antenatal alcohol use, while 30% used tobacco pre- and postnatally. Sample demographic data according to neurodevelopmental domain did not differ significantly from the total sample (***Supplementary Table 1***).

### 4.2 Neurodevelopmental cohort performance cross-sectionally at 5 years

**Figure 4 |:**
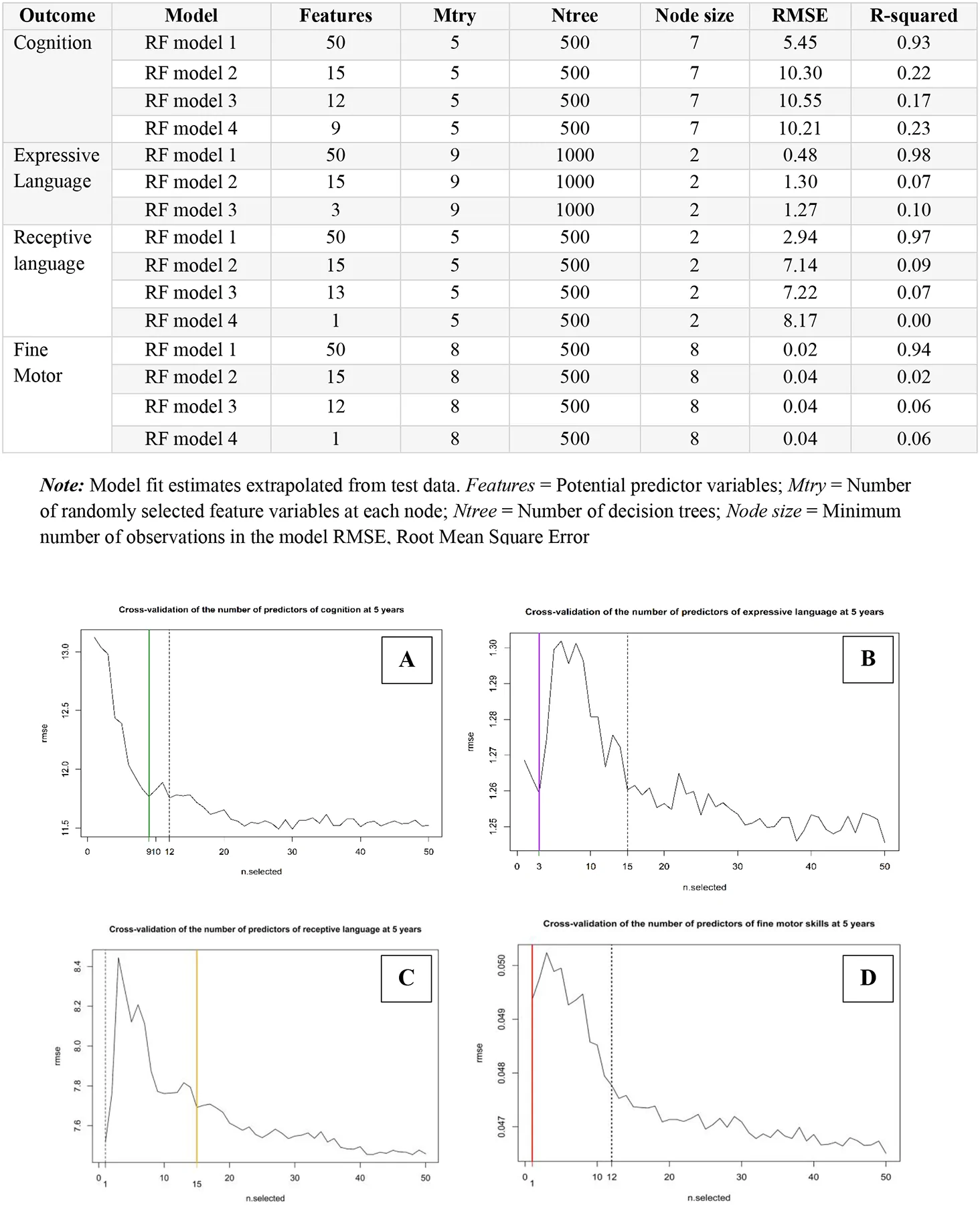
Neurodevelopmental outcomes at 5 years: Model comparison across varying numbers of selected predictors (table), with post hoc 5-fold cross-validation used to identify the optimal predictor set that balances predictive performance and model parsimony. Pane A displays results from cognitive model, pane B displays results from expressive language model, pane C displays 20 results from receptive language model and pane D displays results from fine motor skills model

A series of model comparisons were conducted for each domain to identify the optimal number of top-ranked predictors. This included (1) a model that included all 50 considered predictors, (2) a reduced model retaining the optimal number of predictors identified through cross-validation, in addition to a model (3) that selected the top-ranked 15 predictors (Figure 4). Predictors’ importance was ranked for each domain, based on their mean decrease accuracy. Sensitivity analysis of the imputed data supported the plausibility of the missing at random (MAR) assumption for the vast majority of predictors and outcome variables (***see Supplementary Figure 1***).

#### 4.2.1 Predictors of cognitive outcomes at 5 years

**Figure 5 |:**
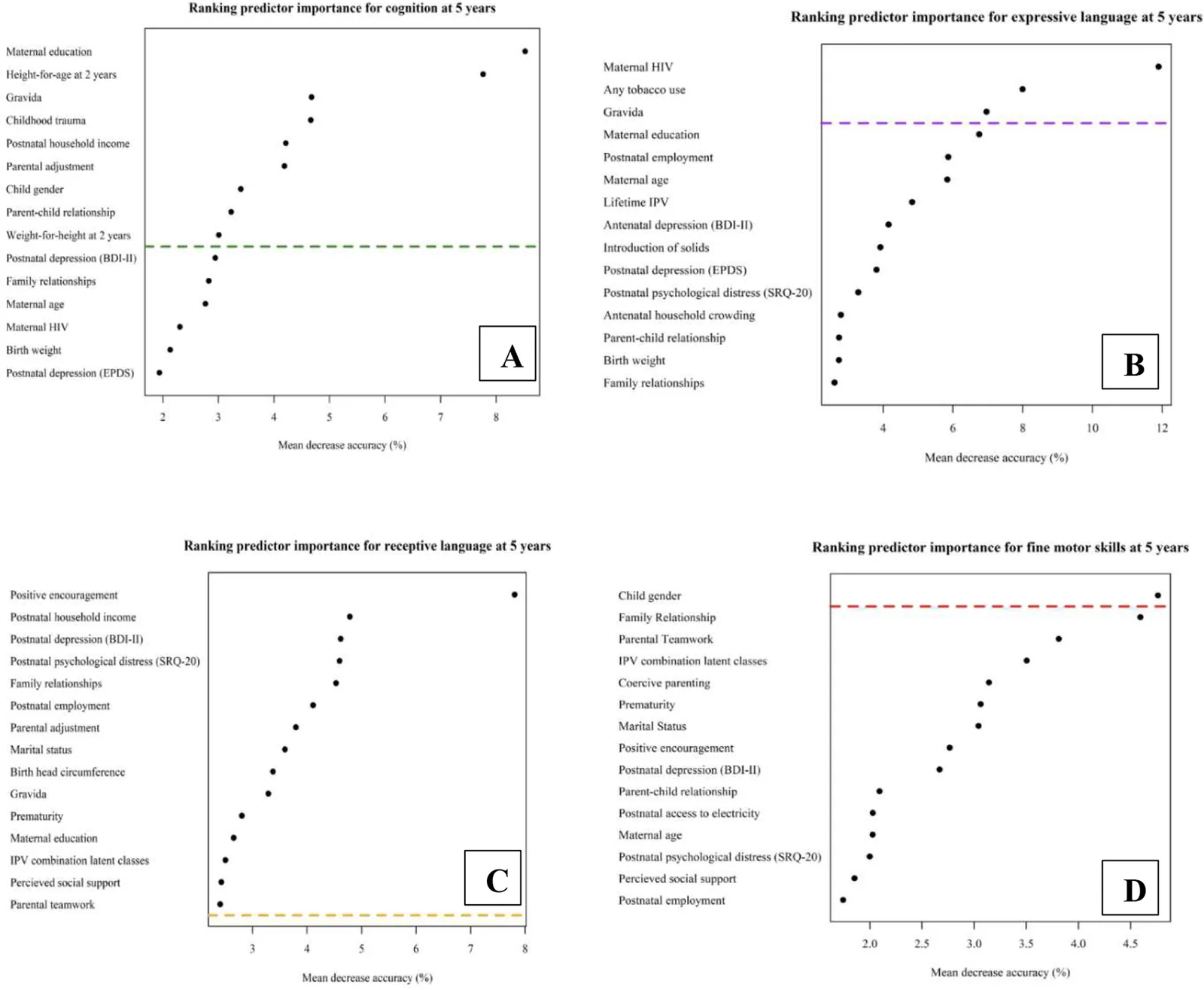
Ranking predictor importance for functional neurodevelopmental domains at 5 years. Results from random forest models displaying the top 15 ranked predictors in terms of importance in predicting outcomes from each of four neurodevelopmental domains at the 5-year timepoint. Predictors’ importance was ranked according to mean decrease accuracy, with predictors ranked from highest to lowest importance. Pane A displays results from cognitive model, pane B displays results from expressive language model, pane C displays results from receptive language model and pane D displays results from fine motor skills model

In a fully specified model of cognition, 93% of total variance in neurocognition at 5 years was explained by the 50 candidate predictors. Prediction error (RMSE) was minimised for a cross-validated model that retained only the top-ranked 9 predictors, (Figure 5), explaining 23% variance in neurocognition. In descending order of importance; these predictors included maternal education, child height at 2 years, maternal gravida, maternal childhood trauma exposure, postnatal household income, parental adjustment, child sex, parent-child relationship and child weight-for height at 2 years.

#### 4.2.2 Predictors of receptive and expressive language outcomes at 5 years

In a model containing all 50 predictors, 98% of variance in expressive language, and 97% of variance in receptive language was accounted for. The cross-validated model retained the 3 top-ranked predictors of expressive language and 15 predictors of receptive language, with models explaining 10% and 7% of variance respectively (Figure 4). The most important predictors of expressive language in rank order, included in-utero HIV exposure, maternal smoking and maternal gravida. Key predictors of receptive language included positive parental encouragement, postnatal household income, postnatal maternal depression, postnatal psychological distress, family relationships, postnatal maternal employment, parental adjustment, maternal marital status, birth head circumference, maternal gravida, child prematurity, maternal education, maternal IPV exposure, maternal perceived social support and reported parental teamwork.

#### 4.2.3 Predictors of fine motor outcomes at 5 years

In a model consisting of all 50 candidate predictors, 94% of variance in fine motor skills at 5 years was explained (Figure 5). Interestingly, a model consisting of 1 vs 12 top-ranked predictors demonstrated equivalent predictive performance, each explaining 6% of variance in fine motor skills. The single-predictor model was preferred for parsimony, with only child sex emerging as the sole relevant predictor of fine motor skills at 5 years highly in predicting receptive language. Child sex was a key factor in the models predicting both cognition and fine motor skills.

### 4.3 Developmental outcome performance trajectories from 2–5 years

**Figure 6 |:**
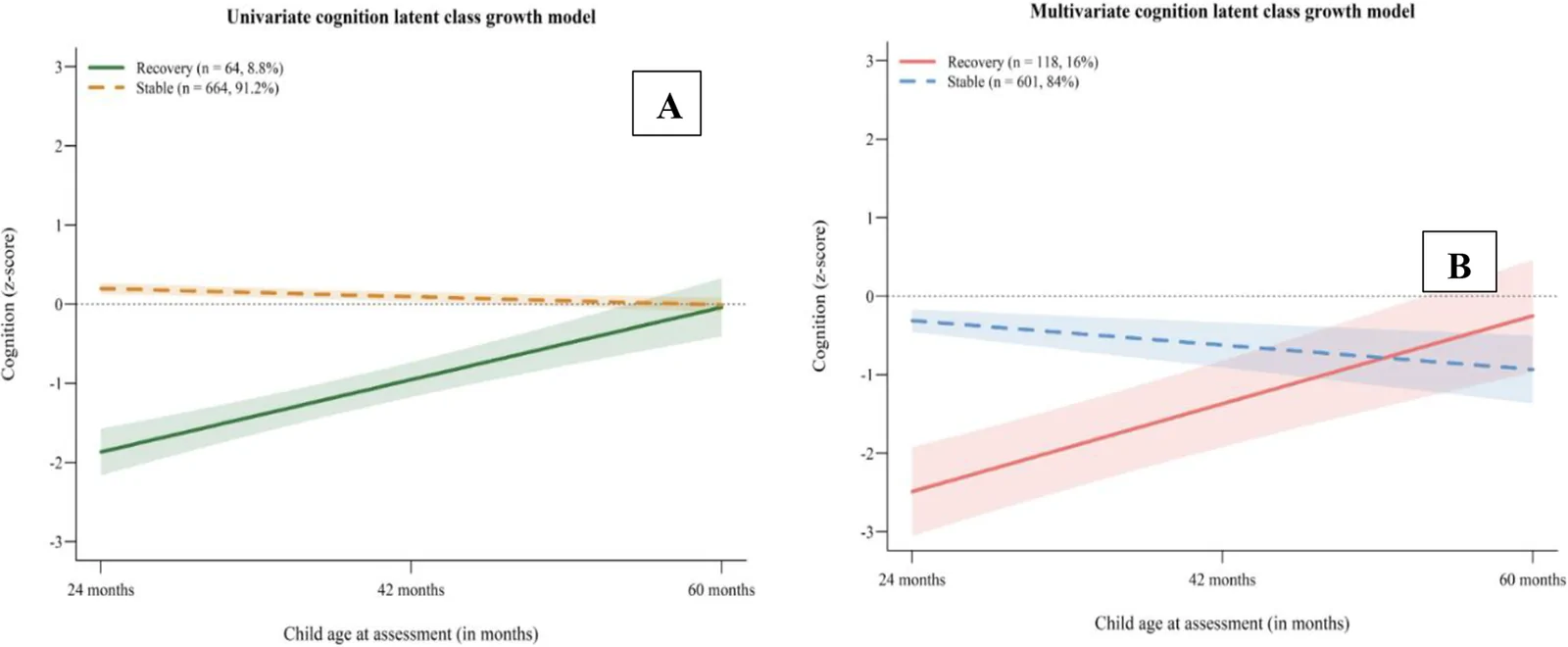
Univariate (Pane A) and multivariate (Pane B) latent class growth modelling (LCGM) of cognition trajectories from 2 to 5 years, where univariate analyses modelled longitudinal measures of cognition, and multivariate analysis jointly modelled longitudinal measures of cognition, alongside expressive language, receptive language, and fine motor skills as indicators of a shared latent developmental process. 95% confidence interval

For univariate cognition LCGM: Two latent growth classes were identified, a: Recovery (N=64, 8.8%, Figure 6) and a stable classes (N=664, 91%, Figure 6), where children in the recovery class initially demonstrated considerably lower cognitive performance at 24 months to those in the stable class, but cognitive performance was comparable by 60 months, whereas the stable class followed a consistent relative cognitive performance trajectory across assessments at 2 years, 3.5 years and 5 years of age. For multivariate LCGM (Figure 6): Two latent growth classes were identified: Recovery (N=118, 16%) and stable classes (N=601, 84%), which showed a largely similar pattern of class membership to that observed in the univariate cognition analysis, particularly for the recovery class, whilst the stable class was less consistent across these three age points. For model comparisons of LCGMs of varying number of latent classes (Table 2). Model selection was based on lowest BIC and highest entropy subject to a minimum class size of 5% or more, where 2 latent classes were selected for both univariate and multivariate neurodevelopmental models. Across models, 64.9% of univariate recovery class members fell within the multivariate recovery class.

#### 4.3.1 Predictors of univariate and multivariate outcomes from 2–5 years

**Figure 7 |:**
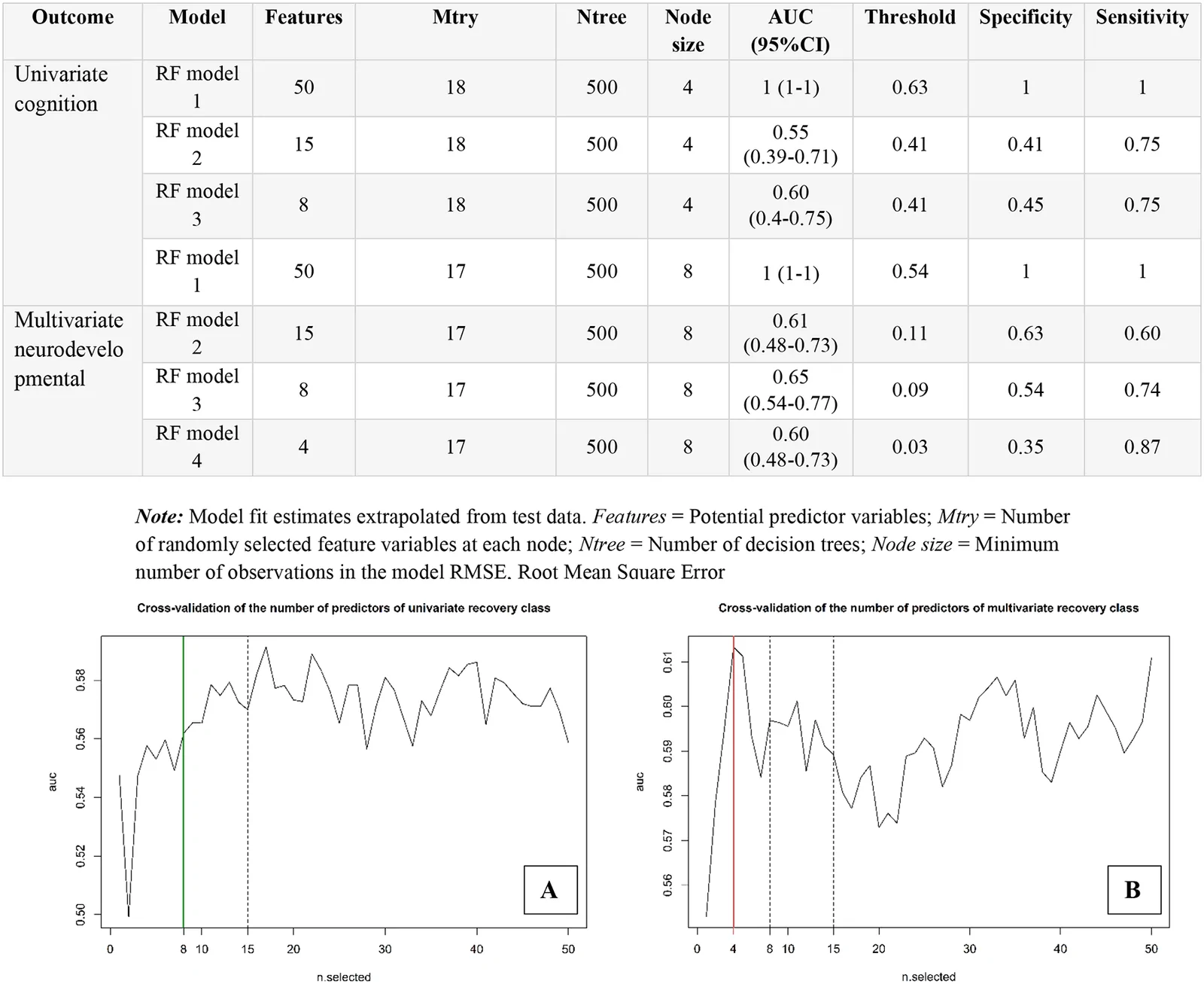
Univariate and multivariate neurodevelopmental outcomes across 2–5 years: Model comparison across varying numbers of selected predictors (Table), with post hoc 5-fold cross-validation used to identify the optimal predictor set that balances predictive performance and model parsimony. Pane A displays results from univariate cognitive model and pane B displays results from multivariate neurodevelopmental model.

Models including all 50 predictors demonstrated perfect discrimination, with AUC = 1, sensitivity and specificity at 1, demonstrating perfect classification of latent classes across both univariate and multivariate models (Figure 7).

**Figure 8 |:**
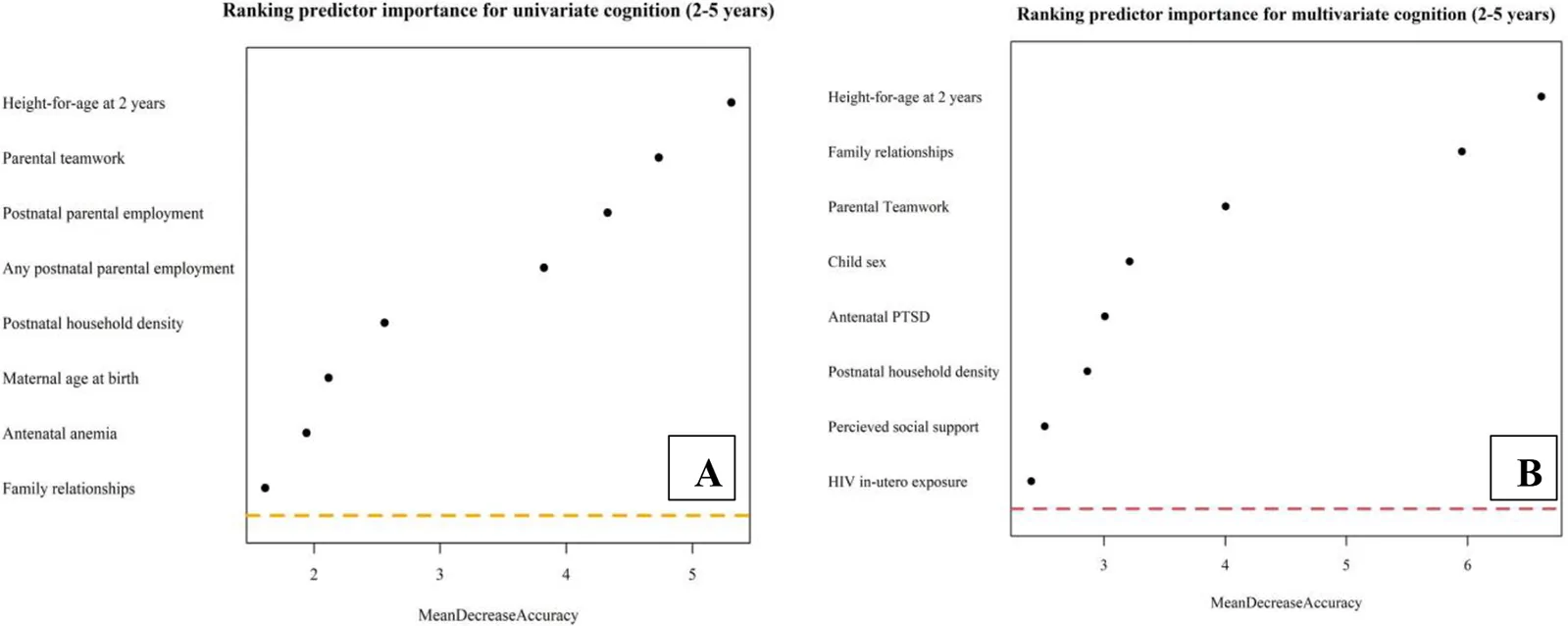
Ranking of predictor importance for Univariate (pane A) and multivariate (pane B) neurodevelopmental outcomes across 2–5 year

Cross-validated models indicated that models retaining the top-ranked 8 predictors achieved optimal predictive performance for recovery latent class membership across both univariate and multivariate models, with higher sensitivity for true recovery class identification (0.75 and 0.74 respectively) compared with specificity for stable class membership (0.45 and 0.54). However, the top-ranked selected predictors were not entirely concordant across univariate and multivariate models. Child height at 2 years, reported parental teamwork, family relationships and postnatal household crowding emerged as important predictors of recovery class membership within both univariate and multivariate models (Figure 8). Predictors for recovery class membership uniquely identified in the univariate neurodevelopmental model included postnatal parental employment, maternal age and antenatal maternal anaemia status, whereas child sex, antenatal maternal PTSD status, maternal social support and in-utero HIV exposure were important in the multivariate model

## 5. Discussion

In this prospective South African birth cohort, early biological, social, psychosocial and family characteristics showed both some common and domain-specific capacity to predict developmental performance at five years. Best fitting, parsimonious random-forest models retained nine predictors for cognition, fifteen for receptive language, three for expressive language and one for fine motor performance. Predictive performance was greatest for cognition, for which the selected model explained 23% of variance; the corresponding models explained 9% of receptive-language variance, 10% of expressive-language variance and 6% of fine-motor variance. These estimates indicate that the available early-life data captured meaningful but incomplete information about later development.

Beyond predictors of neurodevelopmental outcomes at 5 years of age, latent class trajectory analysis revealed developmental pathways between 2–5 years, including a subgroup which achieved catch-up across neurodevelopmental domains (univariate outcome model of cognition as well as multivariate outcome model of cognition, language and fine motor skills). Child height at 2-years of age, parental teamwork and household crowding indices, predicted membership of the recovery class across both univariate and multivariate outcome models. This trajectory modelling showed that low early performance was not necessarily persistent. These methods identify combinations of characteristics that warrant further study and may support risk-enriched developmental monitoring.

Consistent with our earlier findings at 2 years (3), maternal education and household resources, emerged as consistently strong predictors of cognitive and language performance in children at 5 years. Conversely, risks common in our setting, including maternal mental health, exposure to intimate partner violence (IPV), and exposure to maternal HIV all contributed substantially to the model predicting language outcomes at 5 years of age. Specifically, maternal postnatal depression and persistent maternal experience of IPV (reported for both antenatal and postnatal period) were strong predictors of language outcomes. These findings are in keeping with well described hypotheses that psychosocial stress impacts developmental potential through mechanisms such as altered regulation of the hypothalamic-pituitary axis as well as later caregiving capacity (50).

The demonstrated relationship of in utero HIV exposure (HEU) on child development from infancy to age 5 years in the same cohort of children, despite no child HIV infection, is also an important finding. While prior analyses at 2 years in this cohort found subtle differences, our current findings indicate that the gap in certain domains (especially expressive language) may widen with age (51, 52). It is apparent, that even in the era of widespread maternal antiretroviral therapy, careful developmental monitoring and support for children who are HEU remains warranted. Our study adds to global evidence that in utero exposure to HIV, without active infection, may be critical predictors of lasting neurodevelopmental consequences, particularly in environments where such exposure co-occurs with social disadvantage (53).

The broad pattern of factors predicting developmental outcomes in early childhood described here, is consistent with findings from other low- and middle-income country (LMIC) cohorts. A recent pooled analysis of 21 LMIC studies, reported maternal education at secondary school level, to be associated with better cognitive development, whereas preterm birth and early-life nutritional deficits, represented by metrics such as anaemia were linked to poorer cognitive and motor outcomes (54). We found child sex to be the strongest predictor of fine motor skills at age 5 years in our cohort, but other top-ranked predictors that fell short of being included in the optimal model may still be relevant, particularly prematurity. Indicators of in utero growth (specifically head circumference) predicted cognitive language performance. Likewise, social determinants of health have consistently been shown to influence child development across diverse settings. The fact that maternal education and household income remained important predictors of 5-year outcomes in our South African sample reinforces the universality of socio-economic considerations for strategies to support early development. It also suggests that interventions aimed at structural factors such as alleviating poverty and improving parental education could yield developmental benefits comparable to those seen in other global contexts (55).

At the same time, our study addresses a relative gap in the literature by examining some less studied factors in this age range, especially in an African context. Maternal exposure to IPV is not routinely assessed in many cohort studies of child development (54), yet nearly half of the mothers in our study reported exposure to physical, sexual or emotional violence. We found maternal exposure to IPV (particularly emotional and physical violence) as an important factor in the group of predictors of receptive language abilities at age 5 years. Prior research has often focused on child behavioural and emotional outcome-associations of maternal IPV exposure (56), we demonstrate that this exposure strongly contributes to predictive models for linguistic skills at an important age, underscoring the need for awareness about violence prevention and support for affected families as part of early childhood development programs.

Another novel contribution of our work is the focus on maternal lifetime trauma and its intergenerational effects. We observed that maternal childhood trauma in addition to current exposure to violence as reported above, contributed to predictive models for child cognitive outcomes. While data-driven predictive models such as used in this study, don’t allow causal interpretation, the presence of factors which have been demonstrated to explain substantial proportion of the variance observed in developmental outcomes by age 5 years, suggest important contextual considerations when approaching hypotheses for aetiological risk. This aligns with theories of intergenerational transmission of adversity and suggests that a mother’s own early-life environment and mental health may indirectly shape her child’s cognitive trajectory, possibly through impacts on parenting sensitivity, stress regulation, or biological pathways (eg. epigenetic or stress-hormone regulation (57)). Our finding reinforces the need for strategies that address maternal psychosocial wellbeing as part of comprehensive approaches to improve child outcomes.

Our novel analysis of longitudinal developmental trajectories between 2–5 years, revealed a subset of children who had shown delays at 2 years and who demonstrated recovery by 5 years of age. Approximately 9–16% of children fell into this “recovery” class on cognitive and multi-domain developmental trajectories. This potential to examine developmental plasticity and resilience, even among children with the poorest development in infancy, offers a more hopeful perspective compared to strictly cross-sectional approaches. Child-specific factors we found to be associated with membership in the recovery class of the multivariate outcome model included biological sex. Existing evidence in the developmental literature suggests boys may may demonstrate slight developmental disadvantages in high-risk environments and girls may be more responsive to protective inputs (54, 58). Social factors associated with the recovery trajectory included aspects of the postnatal home environment. Household crowding and parental teamwork where both factors which predicted membership of this group. This finding suggests that even for children who were at high developmental risk during infancy, a supportive home context can facilitate recovery ahead of school entry. Although our models were not able to determine direction of effect, at a theoretical level, reduced household crowding might indicate less chaos, more stimulation, or better resource availability for the child, while strong parental teamwork may suggest a more responsive caregiving environment. From a policy perspective, this finding highlights the potential of supporting improved postnatal living conditions and family dynamics to influence childhood developmental potential.

We acknowledge some limitations to this study which include sample attrition over time, potentially biasing results toward families with greater stability. However, the consistency of findings from our 2-year analysis to the 5-year timepoint somewhat mitigates this concern. We further mitigated missing data via imputation, which may have introduced minor biases, but sensitivity analyses suggest robustness. The performance of the multivariate LCGM for neurodevelopment across 4 domains fell below conventional reporting standards (entropy <0.8). However, as this was an exploratory analysis, this limitation was mitigated by high entropy observed in the univariate LCGM model for cognition, (entropy >=0.8), which demonstrated a similar latent group distinction to the multivariate model. Lastly, whilst models were not externally validated using independent data, this limitation was addressed through use of 5-fold internal cross-validation. A key strength of this predictive modelling approach is its ability to identify the most important predictors of neurodevelopment in a complex environment, but we recognise that it does not provide evidence regarding the directionality or causality of these models.

Overall, our findings align with the Nurturing Care Framework promoted by global health initiatives, emphasising five components essential for early development: health, nutrition, security and safety, responsive caregiving, and opportunities for early learning. Many of the factors identified as playing an important role in our predictive models (e.g. violence exposure, maternal HIV, child growth) correspond to deficits in the “security and safety” or “health” components, whereas factors traditionally considered protective (e.g. maternal education, positive parenting environment) facilitate responsive caregiving and early learning (59). Our study provides local evidence reinforcing this framework and highlights components which may best predict early developmental outcomes in an African setting. Over half of the children in our cohort had delays in one or more domains at 2 years of age and although some recovered by 5 years, many did not. Together, these findings highlight the dual importance of considering structural and psychosocial risks as well as family-level strengths in efforts to understand the range of contextual variables which explain a child’s development at a single age or their trajectory across time. This study adds to a growing body of evidence demonstrating the need to consider approaches to understanding child development as the product of both accumulated and interacting influences across educational, economic, anthropometric and family variables. Our contextually informed models aims to inform efforts to improve the odds that vulnerable children both survive and also thrive, entering the school system better equipped to learn and achieve their full potential.

## Supplementary Material

Supplementary Files

This is a list of supplementary files associated with this preprint. Click to download.

- SupplementaryMaterial.docx

## Figures and Tables

**Table 1 |:** Sample demographics

Sample characteristics	Variable reference	Total analytic sample (n=728) | n (%) or mean (sd)
**Child factors**		
***Birth factors***		
Sex	Female	356 (49%)
Prematurity	<37 weeks	104 (14%)
Gravida		1.14 (1.10)
Maternal age at birth	Years	27.28 (5.84)
Birth weight	Kilogram	3.03 (0.58)
Birth head circumference	Centimetre	33.53 (1.98)
***Child Health factors***		
Hospitalisation	Number hospitalised before2 years	177 (24%)
in-utero HIV exposure		167 (23%)
Antenatal maternal anaemia exposure	haemoglobin < 10 g/dl	114 (17%)
***Nutritional and growth factors***		
Exclusive breastfeeding duration	>=5 Months	124 (17%)
Age at start of solid food	Months	4.69 (1.87)
Height-for-age at 24 months	Height-for-age z-score	−1.06 (1.18)
Weight-for-height at 24 months	Weight-for-height z-score	0.32 (1.25)
**Socio-economic factors**		
Marital status	Married/cohabiting	290 (40%)
Maternal education	Completed secondary or higher	271 (37%)
***Employment and income***		
Antenatal maternal employment	Currently employed	182 (25%)
Postnatal parental employment^1^	Currently employed	0.60 (0.36)
Any postnatal parental employment	Currently employed	627 (86%)
Antenatal household income	> R1000 p/m	467 (64%)
Postnatal household income^1^	> R1000 p/m	0.81 (0.23)
Any postnatal household income	> R1000 p/m	715 (99%)
***Resources and household factors***		
Antenatal electricity access	Yes	687 (95%)
Postnatal electricity access^1^	Yes	0.94 (0.17)
Any postnatal electricity access	Yes	717 (99%)
Antenatal running water access	Yes	502 (69%)
Postnatal running water access^1^	Yes	0.76 (0.29)
Any postnatal running water access	Yes	699 (96%)
Antenatal household crowding	No. persons per household	5.13 (2.69)
Postnatal household crowding	Average no. persons per household	5.21 (1.94)
**Maternal mental health factor**s		
***Trauma***		
Childhood trauma	Above threshold	401 (61%)
Antenatal PTSD	Suspected PTSD trauma-exposed trauma unexposed	78 (12%) 80 (12%) 494 (76%)
Antenatal lifetime IPV	Above threshold	306 (47%)
Any postnatal IPV (any recent)	Above threshold	350 (48%)
IPV combination Latent Classes	Class 1: High physical, emotional and sexual IPV Class 2: Decreasing IPV across subtypes Class 3: Moderate emotional & physical IPV and low sexual IPV Class 4: Low IPV	54(8.6%) 71 (11%) 131(21%) 371(59%)
Antenatal psychological distress (SRQ-20)	Above threshold	138 (21%)
Postnatal psychological distress (SRQ-20)^1^	Above threshold	0.09(0.23)
***Depression***		
Antenatal depression (BDI)	Above threshold	123 (19%)
Postnatal depression (BDI)^1^	Above threshold	0.07 (0.22)
Antenatal depression (EPDS)	Above threshold	155 (24%)
Postnatal depression (EPDS)^1^	Above threshold	0.14 (0.27)
EPDS latent classes	Class 1: Mild during pregnancy, slight decrease postpartum Class 2: Mild during pregnancy, increasing postpartum Class 3: Unstable, Peak at 12 months postpartum Class 4: Moderate during pregnancy, minimal postpartum Class 5: Severe during pregnancy and postpartum	543(87%) 38 (6.1%) 14(2.2%) 10(1.6%) 18(2.9%)
***Substance use***		
Antenatal alcohol use	Yes	100(14%)
Antenatal and postnatal tobacco use	No use Prenatal smoking only Postnatal smoking only Pre & postnatal smoking	449 (62%) 14(1.9%) 44(6.1%) 215(30%)
**Parenting & social support**		
Coercive parenting (PAFAS)	Continuous score at 30 months	5.79 (3.65)
Positive encouragement (PAFAS)		1.14 (1.66)
Parent-child relationship (PAFAS)		1.00 (1.70)
Parental adjustment (PAFAS)		2.29 (2.66)
Family relationships (PAFAS)		1.85 (2.47)
Parental teamwork (PAFAS)		1.80 (1.87)
Perceived social support (MSPSS)		5.57 (1.29)

Represented continuously as a proportion^1^. CHEU, children who are HIV-exposed uninfected; PTSD, post-traumatic stress disorder; IPV, intimate partner violence; BDI, Beck Depression Inventory; EPDS, The Edinburgh Postnatal Depression Rating Scale; SRQ-20, The Self reporting Questionnaire; PAFAS, The Parenting and Family Adjustment Scale

**Table 2 |:** Univariate cognition and multivariate neurodevelopmental latent class growth model comparisons

Outcome	Model	No. latent classes	BIC	Entropy	Class 1 (%; PP)	Class 2 (%; PP)	Class 3 (%; PP)	Class 4 (%; PP)
Univariate cognition	LCGM model 1	1	4818	1.00	(100;1)	-	-	-
	LCGM model 2	2	4766	0.84	(8.8;0.83)	(91;0.97)	-	-
	LCGM model 3	3	4785	0.66	(8.9;0.82)	(89;0.88)	(2.1;0.60)	-
	LCGM model 4	4	4793	0.63	(2.74;0.88)	(20.7;0.75)	(71.7;0.82)	(4.8;0.77)
Multivariate developmental	LCGM model 1	1	16182	1.00	(100;1)	-	-	-
	LCGM model 2	2	16052	0.60	(84;0.90)	(16;0.78)	-	-
	LCGM model 3	3	16024	0.58	(41; 0.78)	(56; 0.78)	(2.4;0.88)	-
	LCGM model 4	4	16030	0.63	(82.9;0.81)	(6,1;0.73)	(9.6;0.74)	(1.4;0.79)

***Note:*** BIC = Bayesian Information Criterion; Entropy = Measure of latent class separation; PP = Posterior Probability; LCGM: Latent class growth modelling

## Data Availability

The de-identified data that support the findings of this study are available from the corresponding author upon reasonable request as per DCHS cohort guidelines.

## List of Abbreviations

ASSIST: Alcohol, Smoking and Substance Involvement Screening Test
BDI-II: Beck Depression Inventory, second edition
BSID-III: Bayley Scales of Infant and Toddler Development, third edition
CHEU: Children who are HIV-exposed uninfected
DCHS: Drakenstein Child Health Study
EPDS: Edinburgh Postnatal Depression Scale
HEU: HIV-exposed uninfected
HIV: Human immunodeficiency virus
HREC: Human Research Ethics Committee
IPV: Intimate partner violence
KABC-II: Kaufman Assessment Battery for Children, second edition
LCGM: Latent class growth modelling
LMICs: Low- and middle-income countries
MAR: Missing at random
MCAR: Missing completely at random
mPSS: Modified PTSD Symptom Scale
MSPSS: Multidimensional Scale of Perceived Social Support
NVI: Nonverbal Index
PAFAS: Parenting and Family Adjustment Scale
PPVT-4: Peabody Picture Vocabulary Test, fourth edition
PTSD: post-traumatic stress disorder
RF: Random Forest
RMSE: Root mean squared error
ROC-AUC: Receiver operating characteristic area under the curve
SRQ-20: Self-Reporting Questionnaire, 20-item version
